# External Validation of the KOOS-ACL in the MOON Group Cohort of Young Athletes Followed for 10 Postoperative Years

**DOI:** 10.1177/03635465231160726

**Published:** 2023-04-07

**Authors:** Hana Marmura, Paul F. Tremblay, Dianne M. Bryant, Kurt P. Spindler, Laura J. Huston, Alan M.J. Getgood

**Affiliations:** * Faculty of Health Sciences, Western University, London, ON, Canada; † Fowler Kennedy Sport Medicine Clinic, London, ON, Canada; ‡ Bone and Joint Institute, Western University, London, ON, Canada; § Lawson Research, London Health Sciences Centre, London, ON, Canada; ‖ Department of Psychology, Western University, London, ON, Canada; ¶ Schulich School of Medicine and Dentistry, Western University, London, ON, Canada; # Department of Health Research Methods, Evidence and Impact, McMaster University, Hamilton, ON, Canada; ** Department of Orthopaedics, Cleveland Clinic Florida Region, Weston, Florida, USA; †† Vanderbilt Orthopaedic Institute, Vanderbilt University Medical Center, Nashville, Tennessee, USA; Investigation performed at Western University, London, Ontario, Canada

**Keywords:** knee ligaments, ACL, patient reported outcome, validation, KOOS, young athletes

## Abstract

**Background:**

The Knee injury and Osteoarthritis Outcome Score–Anterior Cruciate Ligament (KOOS-ACL) is a short form version of the KOOS, developed to target populations of young active patients with ACL tears. The KOOS-ACL consists of 2 subscales: Function (8 items) and Sport (4 items). The KOOS-ACL was developed and validated using data from the Stability 1 study from baseline to postoperative 2 years.

**Purpose:**

To validate the KOOS-ACL in an external sample of patients matching the outcome’s target population.

**Study Design:**

Cohort study (diagnosis); Level of evidence, 1.

**Methods:**

The Multicenter Orthopaedic Outcomes Network group cohort of 839 patients aged 14 to 22 years who tore their ACLs while playing sports was used to assess internal consistency reliability, structural validity, convergent validity, responsiveness to change, and floor/ceiling effects of the KOOS-ACL at 4 time points: baseline and postoperative 2, 6, and 10 years. Detection of treatment effects between graft type (hamstring tendon vs bone–patellar tendon–bone) were also compared between the full-length KOOS and KOOS-ACL.

**Results:**

The KOOS-ACL demonstrated acceptable internal consistency reliability (α = .82-.89), structural validity (Tucker-Lewis index and comparative fit index = 0.98-0.99; standardized root mean square residual and root mean square error of approximation = 0.04-0.07), convergent validity (Spearman correlation with International Knee Documentation Committee subjective knee form = 0.66-0.85; Western Ontario and McMaster Universities Osteoarthritis Index function = 0.84-0.95), and responsiveness to change across time (large effect sizes from baseline to postoperative 2 years; *d* = 0.94 [Function] and *d* = 1.54 [Sport]). Stable scores and significant ceiling effects were seen from 2 to 10 years. No significant differences in KOOS or KOOS-ACL scores were detected between patients with different graft types.

**Conclusion:**

The KOOS-ACL shows improved structural validity when compared with the full-length KOOS and adequate psychometric properties in a large external sample of high school and college athletes. This strengthens the argument to use the KOOS-ACL to assess young active patients with ACL tears in clinical research and practice.

The Knee injury and Osteoarthritis Outcome Score (KOOS) is commonly used in clinical research trials, registries, and clinical practice to assess high-functioning patients with anterior cruciate ligament (ACL) tears after ACL injury and reconstructive surgery. However, the KOOS is not specific to ACL injuries, is quite long (42 questions), and may not be as relevant to the young active patients with ACL tears whom we often wish to follow. Additionally, there is no evidence to support the structural validity of the KOOS in patient populations with ACL tears.^2,19^ In a previous study, Marmura et al^8^ conducted analyses in a sample of >600 young (age, 14-25 years) active patients with ACL tears and determined that the KOOS had inadequate structural validity for use in this population. The intended structure of the KOOS— with items assigned to 1 of 5 subscales (Symptoms, Pain, Activities of Daily Living [ADL], Sport and Recreation, and Quality of Life)—was not supported in the sample, and it was evident that major restructuring and/or simplification would be required to establish adequate fit.^8^

Therefore, a short form version of the KOOS was created with the goal of achieving structural validity, removing irrelevant items, and reducing responder burden, while still maintaining adequate psychometric properties. The development of the KOOS-ACL was conducted using data from the Stability 1 study, wherein patients were randomized to hamstring tendon (HT) autograft ACL reconstruction (ACLR) alone or with a lateral extra-articular tenodesis.^3,7^ A preliminary validation of the KOOS-ACL indicated adequate internal consistency reliability, structural validity, convergent validity, and responsiveness to change across time from baseline (preoperative) to postoperative 24 months.^7^ There were some remaining concerns surrounding measurement invariance and ceiling effects starting at 6 months, which, although not unexpected, may influence the ability to detect treatment effects between intervention groups in clinical research. Further studies may be required to identify the ability of the measure to detect change across various follow-up periods and between treatments.

These initial validation analyses were all conducted within the same data set from the Stability 1 study, which presents a lack of external validity and the potential to falsely declare adequate psychometric properties that may not hold outside of the utilized group of patients. Therefore, the purpose of this study was to externally validate the psychometric properties of the KOOS-ACL using the Multicenter Orthopaedic Outcomes Network (MOON) group data set of >800 young athletes (age, 14-22 years) with ACL tears who underwent ACLR with HT or bone–patellar tendon–bone (BPTB) autografts and were assessed at baseline and postoperative 2, 6, and 10 years. It was hypothesized that the KOOS-ACL would show adequate internal consistency reliability, structural validity, convergent validity, and responsiveness to change in this data set of high-functioning patients with ACL tears.

All abbreviations are defined in Table 1.

**Table 1:** Abbreviations Used

ACL	anterior cruciate ligament
ACLR	anterior cruciate ligament reconstruction
ADL	Activities of Daily Living
ANOVA	analysis of variance
BPTB	bone–patellar tendon–bone
CFI	comparative fit index
HT	hamstring tendon
IKDC	International Knee Documentation Committee subjective knee form
KOOS	Knee injury and Osteoarthritis Outcome Score
KOOS-ACL	Knee injury and Osteoarthritis Outcome Score–Anterior Cruciate Ligament
MOON	Multicenter Orthopaedic Outcomes Network
RMSEA	root mean square error of approximation
SRMR	standardized root mean square residual
TLI	Tucker-Lewis index
Tukey HSD test	Tukey honestly significant difference test
WOMAC	Western Ontario and McMaster Universities Osteoarthritis Index

## Methods

### Sample

The MOON group cohort used in this validation study consisted of 839 patients aged 14 to 22 years who experienced an ACL rupture while playing sports and underwent a primary ACLR with HT or BPTB autografts.^18^ This cohort study was approved by the Cleveland Clinic institutional review board (No. 3867). These patients completed the KOOS questionnaire before surgery (baseline) and at postoperative 2, 6, and 10 years.^18^ This sample represents the target population of the KOOS-ACL, young active patients undergoing ACLR, and is similar to that of the Stability 1 study, the sample in which the KOOS-ACL was developed and initially validated. However, the MOON cohort has a younger maximum age (22 vs 25 years) and younger mean age (17.7 vs 19.0 years), as well as longer follow-up time points (10 vs 2 years). Additionally, all patients in the MOON cohort were high school or college athletes, whereas patients in the Stability 1 study had to fit ≥2 of the following high-risk criteria: a pivot-shift grade ≥2, a desire to return to high-risk/pivoting sports, and generalized ligamentous laxity or knee hyperextension >10°.^3,18^ This meant that Stability 1 study participants could be eligible without sport participation, although this was rare.

### External Validation of Psychometric Properties

The KOOS-ACL outcome measure is available in the Appendix (available in the online version of this article). To conduct a rigorous external validation, methodology from the initial validation article was replicated using a data set from an external source (MOON group). Internal consistency reliability, structural validity, convergent validity, responsiveness, floor/ceiling effects, and detection of treatment effects were assessed for the KOOS-ACL, testing our hypotheses at each of the 4 MOON group cohort study time points, using the statistical methods and acceptable thresholds outlined in Table 2. Correlations between the KOOS-ACL and outcome measures assessing similar constructs were conducted to determine convergent validity. KOOS-ACL scores were correlated with the full-length KOOS, International Knee Documentation Committee (IKDC) subjective knee form, and Western Ontario and McMaster Universities Osteoarthritis Index (WOMAC) function scores. The full-length KOOS is a 42-item knee-specific patient-reported outcome measure assessing the 5 subscales of Pain, Symptoms, function in ADL, function in Sport and Recreation, and knee-related Quality of Life.^15^ Domain scores represent the sum of all items in the domain standardized to a score from 0 to 100 (worst to best). The IKDC is an 18-item knee-specific questionnaire querying symptoms, function, and sports activities.^17^ The items are summed and transformed to a score that ranges from 0 to 100 (worst to best). The WOMAC is a 21-item questionnaire widely used to assess symptoms and disability and was originally developed for individuals with hip and knee osteoarthritis.^9^ The function subscale contains 17 items and is scored out of 68. These outcomes measures have all been validated in patient populations of various knee conditions.

**Table 2:** Definitions, Hypotheses Tested, Statistical Methods, and Acceptable Thresholds to Investigate Psychometric Properties of the KOOS-ACL for Validation^*a*^

Psychometric Property	Definition^10^ and Hypotheses	Statistical Method	Acceptable Threshold
Internal consistency reliability	The degree of interrelatedness between items representing 1 construct Hypothesis: unidimensionality of the Function and Sport subscales, indicated by strong internal consistency reliability	Cronbach alpha (α)^20^	.70 < α < .90
Structural validity Measurement invariance across time	The degree to which scores of an outcome measure adequately reflect the dimensionality of the intended constructs measured; a type of construct validity Equal form: the same factor structure is present across time Equal loadings: the same relationships between items and their factors are present across time Equal intercepts: the predicted scores of items can vary across time but at a constant level of the construct Hypothesis: adequate fit indices and change in fit indices reflecting structural validity and measurement invariance from baseline to postoperative 10 y	Confirmatory factor analysis with analysis of fit indices	CFI and TLI > 0.95 RMSEA and SRMR^4^≤0.08 Change in CFI <0.005 and change in RMSEA <0.01 with incremental model constraints
Convergent validity	The degree to which the relationship with another outcome measure intended to measure the same constructs is as hypothesized; a type of construct validity Hypothesis: moderate to strong correlation of subscale and composite scores with the IKDC, equivalent to correlations seen between the full-length KOOS and IKDC; strong correlation with full-length KOOS	Spearman correlation (ρ) with IKDC and WOMAC function scores (owing to nonnormal distribution of scores at some time points)	ρ > 0.60 with IKDC and WOMAC function^11^ ρ > 0.70 with KOOS^11^ *P* < .05
Responsiveness to change across time	The ability of an outcome measure to detect expected change over time in the intended constructs being measured Hypothesis: significant improvement in Function and Sport scores from baseline to postoperative 2 y, with a plateau (nonsignificant change) from postoperative 2 to 10 y; strong correlation between change scores of the KOOS-ACL and full-length KOOS	Repeated measures ANOVA across all time points, with paired *t* tests of subscale scores between consecutive time points, with Cohen *d* effect size^21^ Spearman correlation (ρ) between KOOS-ACL and full-length KOOS mean differences across time These analyses were conducted assuming measurement invariance across time.	*d* > 0.2 *P* < .05 ρ > 0.70 with KOOS mean differences^11^ *P* < .05
Floor and ceiling effects	The proportion of the respondent distribution that will score on the extremes of an outcome measure Hypothesis: no significant ceiling effects at baseline in either score; significant ceiling effects in Function and Sport scores at postoperative 2 to 10 y	Percentage of patients scoring 0 (floor) and 100 (ceiling) points^5^	<15% scores = 100 or 0
Detection of treatment effects	The ability of a measure to detect a treatment effect between groups hypothesized to produce different outcomes Hypothesis: significant differences in subscale scores with minimally small effect sizes between patients who had HT and BPTB autografts at postoperative 2 to 10 y; a significant time × graft type interaction between patients who had HT and BPTB autografts	One-way ANOVA between graft type groups (HT vs BPTB), Tukey HSD test for multiple comparisons, Cohen *d* effect sizes^21^ Mixed ANOVA of repeated measures (graft type = between-patients factor, time = within-patient factor)	*d* > 0.2 *P* < .05

a ANOVA, analysis of variance; BPTB, bone–patellar tendon–bone; CFI, comparative fit index; HT, hamstring tendon; HSD, honestly significant difference; IKDC, International Knee Documentation Committee subjective knee form; KOOS, Knee injury and Osteoarthritis Outcome Score; KOOS-ACL, KOOS–Anterior Cruciate Ligament; RMSEA, root mean square error of approximation; SRMR, standardized root mean square residual; TLI, Tucker-Lewis index.

All analyses were conducted in R Studio using the *lavaan*, *effsize*, *dplyr*, and *psych* software packages.^14,16,21,22^

## Results

### Sample

Patient characteristics are displayed in Table 3. All 839 patients had complete or incomplete KOOS data available at baseline, with an overall rate of 0.8% missingness. Complete baseline KOOS-ACL Function and Sport scores were available for 98% and 94% of patients, respectively. Response rates in the sample were high for the long-term follow-up time points (78%-86%) (Table 3). When *t* tests were run on the main outcome measures to assess for systematic bias based on missingness, there were no significant differences in baseline KOOS-ACL scores—Function (mean difference, 1.3 [95% CI, –2.1 to 3.0]; *P* = .73), Sport (mean difference, 1.6 [95% CI, –3.0 to 6.1]; *P* = .50), or composite (mean difference, 1.0 [95% CI, –2.2 to 4.3]; *P* = .54)—between patients who were missing or not missing scores at postoperative 10 years.

**Table 3:** Patient Characteristics (N = 839)^*a*^

	Mean ± SD	No. (%)
Sex		
Male		443 (52.8)
Female		396 (47.2)
Age, y	17.7 ± 2.2	
Sport at injury		
Basketball		237 (28.2)
Football		205 (24.4)
Soccer		165 (19.7)
Volleyball		38 (4.5)
Baseball/softball		34 (4.0)
Skiing		23 (2.7)
Gymnastics		10 (1.2)
Other		127 (15.1)
Level of sport^*b*^		
High school/college		706 (84.1)
Recreational		132 (15.7)
Graft		
Bone–patellar tendon–bone		539 (64.2)
Hamstring tendon		300 (35.8)
**KOOS-ACL score**		
Function		
Baseline	83.5 ± 15.4	823 (98.1)
2 y	95.1 ± 7.8	722 (86.1)
6 y	95.0 ± 8.4	705 (84.0)
10 y	95.4 ± 7.7	661 (78.8)
Sport		
Baseline	47.9 ± 27.1	787 (93.8)
2 y	84.1 ± 18.2	725 (86.4)
6 y	85.2 ± 19.0	705 (84.0)
10 y	85.9 ± 18.0	654 (77.9)
Composite		
Baseline	65.6 ± 19.2	772 (92.0)
2 y	89.6 ± 12.1	719 (85.7)
6 y	90.1 ± 12.8	699 (83.3)
10 y	90.6 ± 12.0	651 (77.6)

a KOOS-ACL, Knee injury and Osteoarthritis Outcome Score–Anterior Cruciate Ligament.

b Available data: n = 838 (99.9%).

### External Validation of Psychometric Properties

#### Internal Consistency Reliability

Acceptable Cronbach alpha values (.70 < α < .90) indicated reliable KOOS-ACL Function and Sport scores addressing the same underlying construct at all 4 time points (Table 4).

**Table 4:** Cronbach Alpha Values for the KOOS-ACL Subscales Used to Assess Internal Consistency Reliability^*a*^

	Baseline	2 y	6 y	10 y
Function	.89	.85	.87	.85
Sport	.82	.87	.88	.87

a KOOS-ACL, Knee injury and Osteoarthritis Outcome Score–Anterior Cruciate Ligament.

#### Structural Validity and Measurement Invariance

The 2-factor structure of the KOOS-ACL, with 8 Function items and 4 Sport items, was maintained in the MOON cohort as indicated by acceptable fit indices across time (comparative fit index [CFI] and Tucker-Lewis index [TLI] <0.95; root mean square error of approximation [RMSEA] and standardized root mean square residual <0.08) (Table 5). Equal form (structure) across time was confirmed when the 4 time points were evaluated as individual models and as 1 model to evaluate measurement invariance across the time points (Table 6). The Function and Sport factors were related but distinct, as indicated by their correlation at each time point (0.71-0.86). While the correlation at the later time points between factors hovered around the threshold indicated for collapsing factors into 1 (0.85), we thought that the closeness to the threshold coupled with the adequate model fit indices supported the developed KOOS-ACL structure. No modification indices for any individual model suggested that an item would load more highly onto the other factor. When loadings and intercepts of the model were constrained across time, the change in RMSEA was adequately small (<0.01) to confirm measurement invariance; however, the change in CFI was slightly above the acceptable threshold (<0.005) required to confirm equal loadings or intercepts across time.

**Table 5:** Fit Indices of the KOOS-ACL Structure at 4 Time Points Using Confirmatory Factor Analyses to Assess Structural Validity^*a*^

Time Point^*b*^	χ^2^	CFI^*c*^	TLI^*c*^	RMSEA (90% CI)^*d*^	SRMR^*d*^
Baseline	264.47	0.99	0.99	0.07 (0.06-0.08)	0.05
2 y	135.31	0.99	0.99	0.05 (0.04-0.06)	0.04
6 y	176.62	0.99	0.98	0.06 (0.05-0.07)	0.05
10 y	132.26	0.99	0.99	0.05 (0.04-0.06)	0.05

a CFI, comparative fit index; KOOS-ACL, Knee injury and Osteoarthritis Outcome Score–Anterior Cruciate Ligament; RMSEA, root mean square error of approximation; SRMR, standardized root mean square residual; TLI, Tucker-Lewis index.

b Two, 6, and 10 years represent postoperative time points.

c Acceptable fit indices: CFI and TLI >0.95.

d Acceptable fit indices: RMSEA and SRMR <0.08.

**Table 6:** Change in Fit Indices of the KOOS-ACL With Added Constraints Across All Time Points Using Confirmatory Factor Analysis to Assess Measurement Invariance^*a*^

	CFI (Change)	RMSEA (Change)
Equal form	0.937	0.037
Equal loadings	0.925 (–0.012)	0.040 (+0.003)
Equal intercepts	0.896 (–0.029)	0.046 (+0.006)

a CFI, comparative fit index; KOOS-ACL, Knee injury and Osteoarthritis Outcome Score–Anterior Cruciate Ligament; RMSEA, root mean square error of approximation.

#### Convergent Validity

KOOS-ACL scores were strongly and significantly correlated with outcomes assessing similar constructs, including the IKDC (ρ = 0.66-0.85; correlation with composite, function, and sport scores) and WOMAC function (ρ = 0.84-0.95; correlation with function scores), at all 4 time points (Table 7). KOOS-ACL Function and Sport scores were strongly correlated with full-length KOOS-ADL scores (ρ = 0.84-0.94) and Sport and Recreation scores (ρ = 0.86-0.95) from baseline to postoperative 10 years.

**Table 7:** KOOS-ACL Correlation With IKDC and WOMAC Scores to Assess Convergent Validity^*a*^

	IKDC, ρ			WOMAC, ρ
	Composite	Function	Sport	Function
Baseline	0.75	0.72	0.68	0.95
2 y	0.79	0.66	0.76	0.84
6 y	0.83	0.71	0.78	0.87
10 y	0.85	0.71	0.81	0.86

a All Spearman correlations were statistically significant, *P* < .05. IKDC, International Knee Documentation Committee subjective knee form; KOOS-ACL, Knee injury and Osteoarthritis Outcome Score–Anterior Cruciate Ligament; WOMAC, Western Ontario and McMaster Universities Osteoarthritis Index.

#### Responsiveness

**Figure 1.**
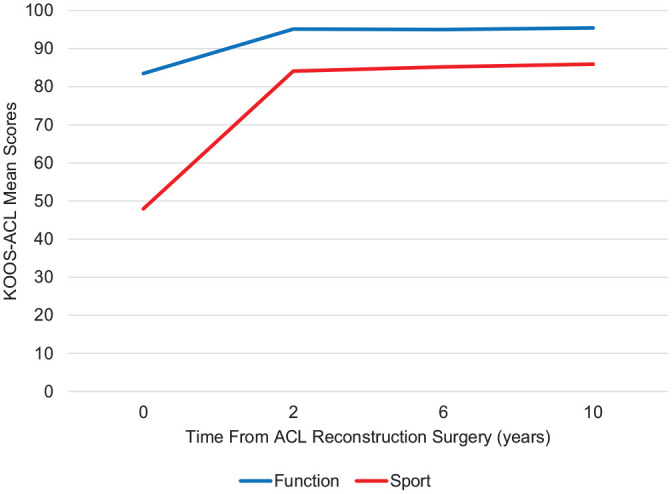
Mean Knee injury and Osteoarthritis Outcome Score–Anterior Cruciate Ligament (KOOS-ACL) scores from baseline to postoperative 10 years in high school and college athletes undergoing ACL reconstruction.

The KOOS-ACL showed adequate ability to detect expected change over time, with a main effect of time on Function (*F* = 252.91; *P* < .001) and Sport (*F* = 604.25; *P* < .001) scores (Figure 1). On the basis of the follow-up time points available in the MOON data set, we hypothesized that we would see a significant increase in scores from baseline to postoperative 2 years, with stable scores from 2 to 10 years after ACLR. Significant improvements with large effect sizes were seen for Function (*d* = 0.94) and Sport (*d* = 1.54) scores from baseline to 2 years, followed by stable mean scores (mean differences <1 point) from 2 to 10 years and negligible effect sizes (Table 8). The mean differences in KOOS-ACL scores across time were highly correlated with mean differences in full-length KOOS scores across time.

**Table 8:** Mean Difference in KOOS-ACL Scores Across Time and Correlation With Mean Difference in Full-Length KOOS Across Time to Assess Responsiveness^*a*^

	Function	Sport
Baseline to 2 y		
Mean difference (95% CI)	11.23^*b*^ (10.07 to 12.39)	35.39^*b*^ (33.09 to 37.70)
Effect size (Cohen *d*)	0.94 (large)	1.54 (large)
Correlation to KOOS mean difference	0.92	0.93
2 to 6 y		
Mean difference (95% CI)	−0.30 (–0.98 to 0.39)	1.18 (–0.34 to 2.70)
Effect size (Cohen *d*)	−0.04 (negligible)	0.06 (negligible)
Correlation to KOOS mean difference	0.83	0.86
6 to 10 y		
Mean difference (95% CI)	0.35 (–0.29 to 0.99)	0.49 (–0.82 to 1.81)
Effect size (Cohen *d*)	0.04 (negligible)	0.03 (negligible)
Correlation to KOOS mean difference	0.79	0.84

a KOOS, Knee injury and Osteoarthritis Outcome Score; KOOS-ACL, KOOS–Anterior Cruciate Ligament.

b Significant difference in scores between time points (*P* < .05).

#### Floor and Ceiling Effects

Less than 15% of patients scored 100 on both KOOS-ACL scores at baseline. Ceiling effects in KOOS-ACL scores were high from postoperative 2 to 10 years, with 46% to 52% of patients scoring 100 on the Function score and 32% to 38% scoring 100 on the Sport score. Similar levels of ceiling effects are seen with the full-length KOOS (45%-53% of patients scoring 100 on the ADLs subscale and 22%-31% scoring 100 on the Sport and Recreation subscale from 2 to 10 years). Floor effects were below the acceptable threshold for all scores and time points.

#### Detection of Treatment Effects

**Figure 2.**
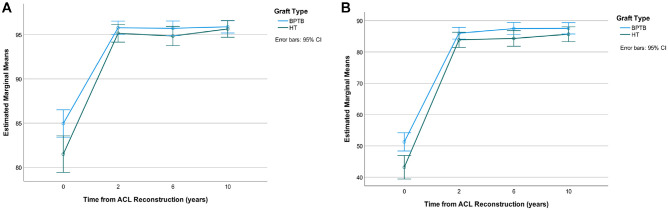
Knee injury and Osteoarthritis Outcome Score–Anterior Cruciate Ligament (ACL) estimated marginal mean scores across time by graft type: (A) Function and (B) Sport subscales. BPTB, bone–patellar tendon–bone autograft; HT, hamstring tendon.

There was a significant difference in baseline scores between groups (HT vs BPTB graft) detected in KOOS-ACL and full-length KOOS scores. When individual analyses of variance (ANOVAs) were conducted to compare groups at each postoperative time point, neither the KOOS-ACL nor the full-length KOOS detected a treatment effect between HT and BPTB autografts in the MOON patient cohort. However, repeated-measures mixed ANOVAs showed a significant interaction between time and graft type (HT or BPTB) in KOOS-ACL subscales, Function (*F* = 3.55; *P* = .03) and Sport (*F* = 3.35; *P* = .03), with consistently lower scores reported by patients who had a hamstring graft (Figure 2). The HT group had significantly lower scores at baseline, and scores became more similar between groups from 2 years on (Figure 2). The same analysis with the full-length KOOS showed a significant time × graft type effect for the Sport and Recreation subscale (*F* = 7.26; *P* < .001) but not the ADL subscale (*F* = 2.85; *P* = .07). The assumption of sphericity was violated for these analyses (ie, variances of the differences among all combinations of related groups are not equal); therefore, *P* values are reported with the Greenhouse-Geisser correction to reduce the likelihood of a type I error.

## Discussion

The most important finding of this study was that the KOOS-ACL was confirmed as a valid and reliable measure to assess the outcomes of this target population, using the MOON group cohort of 839 high school and college athletes followed for 10 years after ACLR. The external validation of the KOOS-ACL strengthens the argument for its use in clinical practice. This study demonstrated that, in addition to its adequate psychometric properties, the KOOS-ACL has improved structural validity and reduced responder burden from that of the full-length KOOS for the target population of high-functioning patients with ACL tears. The similarity of the results between this analysis and the preliminary validation^7^ is evidence of the validity of the new scale. In fact, external validity increases the level of evidence.

This external validation confirms the structural validity of the KOOS-ACL at extended time points of postoperative 6 and 10 years. This is important because the full-length KOOS does not show acceptable structural validity in populations with ACL tears, with significant overlap among subscales/constructs; therefore, we cannot be confident in interpreting the KOOS subscale scores for patients with ACL tears as they are labeled.^8^ We used a combinational rule of thresholds for 4 fit indices to determine structural validity, which reduced the likelihood of making a type I or II error regarding model fit (accepting a model with inadequate fit or rejecting a model with adequate fit, respectively).^1,4^ A strict threshold for comparative fit (CFI and TLI) was used (0.95).^4^ Because the KOOS-ACL subscales contain relatively few items, especially the 4-item Sport scale, a more lenient threshold of absolute fit (standardized root mean square residual and RMSEA) was chosen (<0.08), as these fit indices are more strongly influenced by the number of degrees of freedom in a model.^1^ When all time points were combined, the CFI (0.937) was below the strict 0.95 threshold but still within the range of 0.9 to 0.95, which is generally deemed satisfactory for model fit.^1^

Feasibility is an important psychometric property of patient-reported outcome measures, as evidenced by the diminishing proportions of patients with the required KOOS items available for analysis at subsequent time points (92% at baseline to 78% at postoperative 10 years). The KOOS-ACL is 71% shorter than the full-length KOOS (12 vs 42 items) and is estimated to take <5 minutes for patients to complete. A reduced length is likely to improve feasibility of completion and may help reduce the number of patients lost to follow-up. This may be especially helpful when working with youth athletes who may not be compliant in completing extensive batteries of patient-reported outcome measure questionnaires. In the Swedish knee ligament register, nonrespondents at 2-year follow-up were significantly younger (mean age, 25.9 years) than respondents (mean age, 27.8 years; *P* < .001).^13^ The larger MOON prospective cohort of 3202 patients undergoing ACLR also showed that younger age was a predictor of loss to follow-up, although this relationship was not significant (*P* = .065).^12^

Another key psychometric property of universally utilized outcome measures is cross-cultural validity. Because the KOOS-ACL includes a subset of the KOOS items with identical phrasing, the previously completed translation and cross-cultural validation studies apply to the KOOS-ACL as well. The English and Swedish versions of the KOOS were developed concurrently, and translated versions are available in Austria-German, Chinese, Croatian, Czech, Danish, Dutch, Estonian, French, German, Greek, Hindi (India), Italian, Japanese, Korean, Latvian, Lithuanian, Norwegian, Persian, Polish, Portuguese, Russian, Singapore English, Slovakian, Slovenian, Spanish (Peru), Spanish (United States), Thai, Turkish, and Ukrainian.^6^

Because the KOOS-ACL was designed for a very specific target population—young active patients with ACL deficiency—it was important that validation be conducted within a data set of patients matching this profile, such as the MOON group young athlete cohort. The Stability and MOON patient samples are very similar in terms of age (mean years [range], 19.0 [14-25] vs 17.7 [14-22]) and sport participation.^3,18^ Interestingly, the MOON and Stability groups showed nearly identical scores for baseline Function (83 vs 82), 2-year Sport (95 vs 96), and 2-year Function (85 vs 85).^7^ The results of the 2 validation studies also show strikingly similar structural validity fit indices, correlations with IKDC scores, and Cronbach alpha values of internal consistency reliability.^7^ The likeness of scores and psychometric properties reported from these 2 cohorts indicates consistency in KOOS-ACL assessment of high-functioning patients with ACL tears. The MOON group cohort did have a notably higher mean baseline sport score as compared with the Stability sample (48 vs 38). This may reflect the fact that all patients in the MOON group cohort were athletes and injured while playing sports.^17^ In the Stability 1 study, playing a competitive pivoting sport was only 1 of several possible high-risk inclusion criteria, but even those who did not meet this criterion were highly active.^3^ The correlation between the subscales (Function and Sport) appeared to increase at later time points (postoperative 2-10 years). This could reflect that individuals are participating in lower levels of sport with increasing age; therefore, the constructs of Function and Sport start to become more similar.

In addition to the necessary step of validating the KOOS-ACL in an external data set, this study introduces new follow-up time points (6 and 10 years after ACLR) and a new graft type for ACLR (BPTB), which widens the applicability of the KOOS-ACL within the target population. Prominent ceiling effects are evident at these later follow-up time points, which may limit the usefulness of the KOOS-ACL in detecting treatment effects between intervention groups. However, it is not surprising that a large proportion of high school and college athletes are doing functionally well years after ACLR, as this is the desired and expected outcome of surgical intervention. More sensitive outcome measures, such as difficult performance-based or functional tests, may be needed and more appropriate to detect intervention effects, if they exist, at later time points. Importantly, the internal consistency reliability, structural validity, and convergent validity of the KOOS-ACL did not degrade below any of the acceptable thresholds after the 2-year time point assessed in preliminary validation. KOOS-ACL scores showed stability from postoperative 2 to 10 years, as hypothesized for this patient population. It may make sense to transition from the KOOS-ACL to full-length KOOS at later time points (>10 postoperative years), when symptoms of osteoarthritis may appear and items from the full-length KOOS assessing lower levels of function may be relevant. On the basis of this external validation study, we believe that the KOOS-ACL can be appropriately utilized for high-functioning patients with ACL tears until postoperative 10 years.

Although the KOOS-ACL did not elucidate any treatment effects between graft types in this cohort (despite known differences in resultant graft failure risk), the fact that similar results in the ANOVAs were seen with either the KOOS-ACL or the full-length KOOS indicates criterion validity of the short form. Additionally, the KOOS-ACL Function subscale was able to detect a significant time × graft type interaction, which was not seen in the full-length KOOS ADL subscale. This interaction may be driven by the lower baseline scores in patients who had a hamstring autograft reconstruction. Graft choice in this patient cohort was based on patient/surgeon decision (not randomized). A previous study showed that of 17 participating surgeons, 4 almost always chose BPTB, 4 almost always chose HT, and the remaining 9 switched between graft choices depending on their personal algorithms.^18^ It also showed that patients may have been more likely to receive a BPTB graft if they were younger, had high-grade laxity, played organized high school or college sports, had a lower body mass index, or were Black.^18^ It is not clear whether these characteristics would be linked to higher baseline scores on the KOOS.

The external validation is an important step in creating a patient-reported outcome measure. The KOOS-ACL has now been assessed and validated in >1400 young active patients with ACL tears. However, both validation studies to date have included North American patient cohorts. Further validation would be beneficial to increase our confidence in using the KOOS-ACL worldwide for high-functioning patients with ACL tears. The KOOS-ACL can be tested in any data set that has full-length KOOS data available.

## Conclusion

The KOOS-ACL shows improved structural validity as compared with the full-length KOOS and adequate psychometric properties in a large external sample of high school and college athletes. This strengthens the argument to use the KOOS-ACL to assess young active patients with ACL tears in clinical research and practice.

## Supplemental Material

sj-pdf-1-ajs-10.1177_03635465231160726 – Supplemental material for External Validation of the KOOS-ACL in the MOON Group Cohort of Young Athletes Followed for 10 Postoperative Years Click here for additional data file. Supplemental material, sj-pdf-1-ajs-10.1177_03635465231160726 for External Validation of the KOOS-ACL in the MOON Group Cohort of Young Athletes Followed for 10 Postoperative Years by Hana Marmura, Paul F. Tremblay, Dianne M. Bryant, Kurt P. Spindler, Laura J. Huston and Alan M.J. Getgood in The American Journal of Sports Medicine
