# Supplementary material for: External Validation of the KOOS-ACL in the MOON Group Cohort of Young Athletes Followed for 10 Postoperative Years
Source: Am J Sports Med. 2023 Apr 7;51(6):1457–65. doi: 10.1177/03635465231160726 (PMC10155281; doi:10.1177/03635465231160726)
Supplement: sj-pdf-1-ajs-10.1177_03635465231160726 – Supplemental material for External Validation of the KOOS-ACL in the MOON Group Cohort of Young Athletes Followed for 10 Postoperative Years [file sj-pdf-1-ajs-10.1177_03635465231160726.pdf]

KOOS-ACL

Instructions:
Answer every question by checking the appropriate box, only one box for each question. If you are unsure about how to answer a question, please give your best answer.

Function

1. How severe is your knee joint stiffness after first waking in the morning?

None	Mild	Moderate	Severe	Extreme

2 to 8. The following questions concern your physical function. By this we mean your ability to move around and to look after yourself. For each of the following activities, please indicate the degree of difficulty you have experienced in the last two weeks due to your knee.

	None	Mild	Moderate	Severe	Extreme
Descending Stairs					
Standing					
Bending to floor/picking up an object					
Walking on flat surface					
Lying in bed (rolling over, maintaining knee position)					
Sitting					
Light domestic duties (cooking, dusting, etc.)					

Sport

1 to 3. The following questions concern your physical function when being active on a higher level. The questions should be answered thinking of what degree of difficulty you have experienced during the last two weeks due to your knee.

	None	Mild	Moderate	Severe	Extreme
Running					
Jumping					
Twisting/pivoting on your knee					

4. How much are you troubled with lack of confidence in your knee?

Not At All	Mildly	Moderately	Severely	Totally

Scoring

Individual question items are scored as 0 to 4 (from “None” to “Extreme”, respectively (Function q1-8 and Sport q1-3), or “Not At All” to “Totally”, respectively (Sport q4)).

The KOOS-ACL Function and Sport scores can be calculated in the same way as full-length KOOS subscale scores.

The following equivalent equations can be used to calculate Function and Sport scores:

$$100 - \left(\frac{sum\ of\ subscale\ items * 100}{maximum\ subscale\ score}\right)$$

OR

$$100 - \left(\frac{average\ of\ subscale\ items}{4} * 100\right)$$

Function and sport scores should be calculated, assessed, and analyzed individually.

A total score should not be calculated.

A composite score can be calculated by averaging the two subscale scores, but comprehensive psychometric properties of this score have not been assessed.
